# Cerebro-Spinal Meningitis

**Published:** 1865-03

**Authors:** F. R. Payne

**Affiliations:** Marshall, Illinois


### CEREBRO-SPINAL MENINGITIS.

BY F. R. PAYNE, M.D., Marshall, Illinois.

This epidemic, usually called "spotted fever," has for the last five months prevailed to an alarming extent in this community. It is a terrible disease, and in my opinion, based upon limited observation, if treatment is delayed for twelve hours, death is inevitable.

The disease is ushered in by a chill, or rather tremor, surface of the body cold, pulse, if at all perceptible at the wrist, small and slow, the skin an ash or light lead-color, pain in the head, back, or some of the extremities, a tremulous and painful sensation in the epigastrium. In a short time the eyes become suffused, pupils dilate; the patient becomes restless, and very sensitive.

There is all the evidence of congestion and depression, clearly indicating some toxic agent in the blood, which seems to have a strong affinity for the brain and spinal marrow. The blood is rapidly disorganized—its vitality destroyed—the capillaries and vessels of the brain lose their contractility, as shown by deep moaning, petechical eruptions over the surface of the body. At this stage the patient is partially delirious, but in most cases they will answer questions correctly. The bowels, in nearly all cases, are constipated, tongue slightly coated, and in most of them we find contraction of the posterior muscles of the neck.

Death frequently takes place in five or six hours after the first symptoms appear. I agree with Dr. McCoy, of Morgan County, Illinois; his observation corresponds with what is presented in this locality. The circulation gives out first, then the intellect, and lastly, respiration. Without prompt and energetic treatment, the eyes are frequently closed in death before reaction. In a few hours after the chill, the congestion and depression will completely exhaust the vital powers of the system, and remedial agents will accomplish nothing, even if reaction is established.

In Clark County, we have on former occasions been visited with epidemics in many respects similar to the one now raging. In 1845–46, Dr. James Smick and I met with a terrible and fatal epidemic, vulgarly called "*black tongue*," but, as shown by *post mortem* examinations, was cerebro-spinal arachnitis. This disease first made its appearance in the cuticle, and in all respects resembled ordinary erysipelas, but soon extended through the mucous membranes to internal parts, and then we had symptoms almost precisely the same as now found in *spotted fever*—congestion, depression, and partial delirium. We then, on account of the epidemic prevailing in wet or damp weather, favorable for intermittents, supposed that the toxic agent was *malaria*. We are now thoroughly convinced that the blood-poison is not the same as that which produces intermittent and remittent fevers. In 1845–46, *post mortem* examinations revealed congestion of the bloodvessels of the brain and spinal marrow, and, in the language of Dr. Smick, "the arachnoid was inflamed in every part of it, and covered with a thick layer of pus;" but still we had great depression, contra-indicating the resort to antiphlogistic remedies. It is evident that malaria is not the origin of this class of diseases, from the fact that we have it in districts almost entirely exempt from the prevalence of malaria, and its absence in localities where the generator of intermittents is known to exist. I learn from Dr. J. D. MITCHELL, who located in Darwin, Clark County, after SMICK and PAYNE left that place, that in 1858, a similar and very fatal epidemic prevailed to an alarming extent in that locality, which he denominated *erysipelas of the brain.* In all of these cases the symptoms were nearly the same as now in spotted fever. They had no premonitory external symptoms, as in 1845, of the approach of a terrible disease. The malady was ushered in with a chill, or rigor, great depression, and congestion of the brain and spinal marrow. This epidemic prevailed in that locality only, and all the cases were in the vicinity of Darwin, on the Wabash River; no cases above or below that point. In this county we had no visitation of an epidemic of this character until the winter and spring of 1863–64; but we learn from Drs. S. JUMPER and CHARLES GORHAM, that what is now called *spotted fever* then prevailed to a fearful extent in the localities of Darwin and York, in the south part of Clark and north portion of Crawford County.

In November, 1864, we were, in this locality, visited with the terriffic disease called "*spotted fever*, and from that time until present writing an element has existed in the atmosphere, or somewhere else, which has caused an extraordinary amount of eruptive fevers. In ordinary cases of pneumonia, we usually have petechia or wine-colored spots, frequently all over the body; and in March, 1865, many unmistakable cases of small-pox made their appearance.

It has this winter and spring been clearly demonstrated that, when eruptive diseases prevail in a community as an epidemic, the virulence of small-pox contagion is at least sharpened; but we have no positive evidence of it existing epidemically. We do not believe that the epidemic influences which produce these eruptive fevers are capable of generating small-pox.

Within the last five months I have treated thirty-one cases of what is called spotted fever. Fourteen have died; the others are all well except one, a little child, two years old. It seems tolerably well, but sight and hearing are much impaired. My treatment after the first cases was based upon the views and observations of Dr. McVey. When this disease is first ushered in, it is important, and absolutely necessary, that we resort promptly and energetically to the use of arterial or general stimulants. Under their influence the heart and arteries pulsate with more vigor, and the contracted capillaries become distended with red blood, and all the functions, especially those of the nervous system, are rendered more active. For this purpose we use brandy or good whiskey freely. In a disease so rapid in its march and terrific in its nature, it is necessary to use, in addition to these remedies, agents which will act directly on particular organs or apparatus. The capillaries and vessels of the brain are congested, and opium is perhaps the best remedy known to fill the important indication of arousing them to action, that they may rid themselves of the morbid accumulation of fluid in their structure. Quinine is certainly contraindicated, and in all my recent cases I have used arsenic as an anti-periodic. In the treatment of thirty-one cases fourteen died, and it is unfortunate that I was not able in any of the cases to procure a *post mortem* examination. Drs. R. F. Williams and Rains also lost a number of cases.

After the first few cases, our treatment was, in the chill, brandy, mustard to spine, legs, and arms; opium to adult in 3 to 5 grain doses, every three or four hours; hot rocks, and stimulating friction of the surface. If reaction could thus be established, we diminished the brandy and continued opium, to which was added alterative doses of hydrargyri chloridi mite, and moderately keeping the bowels open with mild aperients. Alternately with the opium powders, we give four to six drops of Fowler's Solution.

This treatment is certainly the most successful, and we feel confident that if early adopted, a majority of the cases will recover.

